# Low Hemoglobin Level and Elevated Inflammatory Hematological Ratios Associated With Depression and Sleep Disturbance

**DOI:** 10.7759/cureus.56621

**Published:** 2024-03-21

**Authors:** Tetsuya Akaishi, Kumi Nakaya, Naoki Nakaya, Mana Kogure, Rieko Hatanaka, Ippei Chiba, Sayuri Tokioka, Satoshi Nagaie, Soichi Ogishima, Atsushi Hozawa

**Affiliations:** 1 Department of Education and Support for Regional Medicine, Tohoku University Hospital, Sendai, JPN; 2 Division of General Medicine, Tohoku University Hospital, Sendai, JPN; 3 Department of Preventive Medicine and Epidemiology, Tohoku Medical Megabank Organization, Tohoku University, Sendai, JPN; 4 Division of Epidemiology, School of Public Health, Tohoku University Graduate School of Medicine, Sendai, JPN; 5 Division of Personalized Prevention and Epidemiology, Tohoku University Graduate School of Medicine, Sendai, JPN; 6 Department of Informatics for Genomic Medicine, Tohoku Medical Megabank Organization, Tohoku University, Sendai, JPN

**Keywords:** sleep disturbance, platelet-to-lymphocyte ratio (plr), neutrophil-to-lymphocyte ratio (nlr), hemoglobin level, depressive state

## Abstract

Background: The relationship between blood cell profiles, including hemoglobin (Hb) levels and inflammatory hematological ratios, and mental health problems currently remains unclear.

Aim: This study aimed to investigate the relationship between blood cell profiles and mental health issues, including depressive state and sleep disturbance, while adjusting for potential demographic confounders.

Methodology: This retrospective, cross-sectional, observational study used a population-based medical database from the Tohoku Medical Megabank Project with more than 60,000 volunteers. Data on age, sex, daily tobacco use, body mass index, and self-reported scores on the Kessler Psychological Distress Scale (K6), Athens Insomnia Scale (AIS), and the Center for Epidemiologic Studies Depression Scale (CES-D) were collected.

Results: A total of 62,796 volunteers (23,663 males and 39,133 females), aged ≥20 years at the time of the blood test, agreed to participate in this study. Among the evaluated blood cell profiles, Hb, hematocrit, neutrophil-to-lymphocyte ratio (NLR), and platelet-to-lymphocyte ratio (PLR) were significantly correlated with the K6, AIS, and CES-D scores, with strong statistical significance (p<0.0001 for all) in bivariate correlation analyses. A significant adjusted odds ratio (aOR) of the Hb level for elevated CES-D scores (aOR=0.965 [95% CI: 0.949-0.981], p<0.0001) was confirmed after adjusting for demographic data and daily tobacco use using a logistic regression model. Sensitivity analyses revealed that these associations existed in both males and females but were more prominent in the former. In male participants, a low Hb level was significantly associated with an elevated AIS score. The evaluated inflammatory hematological ratios, including NLR, PLR, and monocyte-to-lymphocyte ratio (MLR), also showed significant aORs with the K6, AIS, and CES-D scores after adjusting for demographic background.

Conclusion: Low Hb levels and elevated inflammatory hematological ratios (NLR, MLR, and PLR) were associated with depressive state and sleep disturbances in the general population.

## Introduction

A possible relationship between chronic inflammation and psychiatric disorders, including mood disorders and schizophrenia, has been proposed in recent years [1,2]. However, the exact relationship between circulating blood cell profiles and mental health problems such as depressive state and sleep disturbance in the general population remains uncertain. As one of the blood cell profiles, low hemoglobin (Hb) levels have been reported to negatively impact a wide spectrum of psychiatric disorders such as depression and cognitive functions [3-8]. Hb levels are influenced by many factors such as sex, age, and smoking status [9]. Because collecting comprehensive data from a large number of individuals is difficult, the exact relationship between low Hb levels and mental disturbances in the general population remains uncertain. As most patients with anemia in the general population can be treated with oral medications, determining the risk of low Hb levels for mental disturbances is highly important. Moreover, the impact of other blood cell profiles such as white blood cell (WBC) count, WBC subpopulations, platelet count, and derivative inflammatory hematological ratios on mental health remains largely unevaluated. Therefore, this study utilized a large population-based medical database and analyzed the relationship between blood cell profiles, including the Hb levels and inflammatory hematological ratios, and mental health issues, while adjusting for potential demographic confounders.

## Materials and methods

Study design

**Figure 1:**
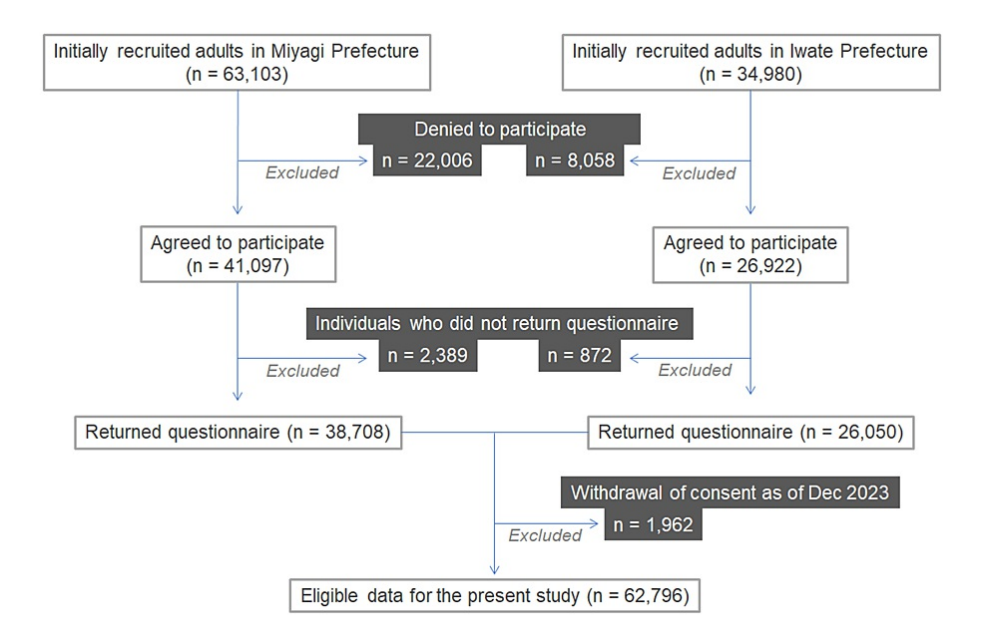
Flow diagram of the study design From the initially recruited adults aged 20–74 years, those who did not return the questionnaire or withdrew the consent to participate as of Dec 2023 were excluded from the subsequent analyses.

This retrospective, cross-sectional, observational study used a population-based medical database from the Tohoku Medical Megabank Organization (ToMMo) and Iwate Medical Megabank Organization (IMM) Cohort Study Projects in Japan [10]. The flow diagram of the study design is shown in Figure 1. The participants lived in the Miyagi and Iwate Prefectures and volunteered to participate in the project. All participants were enrolled between April 2013 and March 2016, and their ages at enrollment were from 20 to 74 years. Older adults aged ≥75 years were not recruited. Residents who visited the recruitment centers to receive health checkups at 28 municipalities in Miyagi Prefecture and 18 municipalities in Iwate Prefecture were recruited. Among the approximately 100,000 individuals who were requested to participate, more than 60,000 agreed to offer their medical information for research purposes. From them, those who did not return the questionnaire or withdrew the consent to participate as of Dec 2023 were excluded from the subsequent analyses.

Collected variables

Data on age, sex, body mass index (BMI), and current daily tobacco use were collected from all participants. Age, BMI, and daily tobacco use were used as continuous variables, whereas sex was used as a nominal variable. The daily amount of tobacco use among current nonsmokers was set at 0. As mental health-related measures, the scores of the Kessler Psychological Distress Scale (K6), Athens Insomnia Scale (AIS), and Center for Epidemiologic Studies Depression Scale (CES-D) were collected. The K6 score ranged from 0 to 24, and it was used to measure nonspecific psychological distress [11]. The AIS score ranged from 0 to 24, and it was used to evaluate sleep disturbance [12]. The CES-D score ranged from 0 to 60, and it was used to measure depressive symptoms [13]. The cutoff level to decide the presence of disturbance was K6 score ≥13, AIS score ≥6, and CES-D score ≥16 [11,13-15]. For the blood cell profiles, the overall WBC, lymphocyte, monocyte, eosinophil, basophil, neutrophil, red blood cell (RBC), and platelet counts were collected. Derivatives from blood cell counts, such as the monocyte-to-lymphocyte ratio (MLR), neutrophil-to-lymphocyte ratio (NLR), and platelet-to-lymphocyte ratio (PLR), were collected.

Statistical analysis

Distributions of continuous variables are described as medians and interquartile ranges (IQR; 25-75 percentiles). Quantitative data were compared between the two groups using the Mann-Whitney U test. Correlations between two quantitative variables were evaluated with Spearman’s correlation coefficients (ρ). Using the aforementioned cutoff level for each mental health-related index, logistic regression analyses were performed with explanatory variables, including each focused blood cell profile (including Hb, NLR, MLR, and PLR). In addition to blood cell-related laboratory data, age, sex, BMI, and daily tobacco use were used as explanatory variables in the logistic regression models. The adjusted odds ratio (aOR) was reported with IQR. A p-value threshold of 0.05 in bivariate analyses and of 0.01 in subsequent multivariable analyses was used to decide the statistical significance. The bivariate correlation coefficients were measured with an aim to explore the potential confounders to be included in the subsequent multivariable analyses, and the alpha level was not adjusted for multiple comparisons. The subsequent multivariable analyses were of confirmatory nature, and the alpha level was adjusted for the multiple comparisons in each subgroup analysis. All statistical analyses were performed with JMP Pro version 17 (SAS Institute, Cary, NC, USA).

Ethics

This study was approved by the Institutional Review Board of the Tohoku University Graduate School of Medicine (approval number: 2021-1-791). Written informed consent was obtained from all participants. The process of this study was performed in accordance with the latest version of the Declaration of Helsinki, as revised in 2013.

## Results

Participants

A total of 62,796 individuals (23,663 males (38%) and 39,133 females (62%)) fulfilled the inclusion criteria and were evaluated in the subsequent analyses. The median age at enrollment was 64 years (IQR 56-69 years). Valid scores without missing sub-scores were obtained from 60,951 individuals (97%) for the K6 scores, 61,304 individuals (98%) for the AIS scores, and 59,099 individuals (94%) for the CES-D scores. The K6 scores were significantly lower in males than in females (median: 3 [IQR 1-6] vs. 4 [IQR 1-7], p<0.0001; Mann-Whitney U test). The AIS scores were significantly lower in males than in females (median: 2 [IQR 1-4] vs 3 [IQR 1-6], p<0.0001). The CES-D scores were slightly lower in males than in females (median: 12 [IQR 9-15] vs 12 [IQR 9-17], p<0.0001).

Correlations between mental health scores and other variables

**Figure 2:**
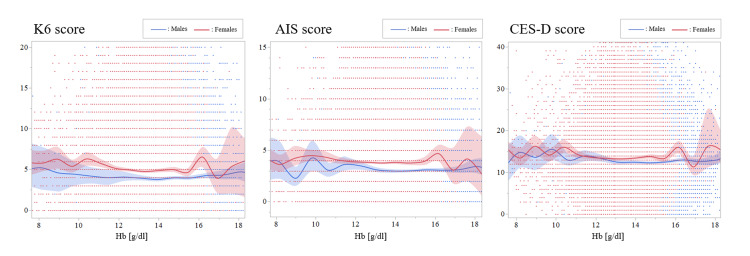
Relationships between the hemoglobin and mental disturbance levels Gradual declines in K6, AIS, and CES-D scores, in parallel with an increase in hemoglobin levels, were observed, especially in male participants. K6, Kessler Psychological Distress Scale; AIS, Athens Insomnia Scale; CES-D, Center for Epidemiologic Studies Depression Scale

Next, Spearman’s correlation coefficients (ρ) between each of the three mental health indices and the other evaluated demographic and laboratory variables were calculated (Table 1). Although the obtained effect sizes were relatively small for all investigated variables, age, daily tobacco use, and BMI were significantly correlated with the mental health indices. Among the evaluated blood cell-based laboratory data, Hb and hematocrit levels showed the most significant correlations with mental health indices (p<0.0001 for all pairs). The scatterplots between Hb levels and scores for each of the three mental health-related indices are shown in Figure 2, together with the approximation lines and confidence interval areas.

**Table 1:** Spearman’s correlation coefficients between each mental disturbance score and other variables among the overall participants The Spearman’s ρ was calculated with the p-value for each pair of the variables. K6, AIS, and CES-D scores were used as continuous variables. AIS, Athens Insomnia Scale; BMI, body mass index; CES-D, The Center for Epidemiologic Studies Depression Scale; K6, Kessler Psychological Distress Scale; MLR, monocyte-to-lymphocyte ratio; NLR, neutrophil-to-lymphocyte ratio; PLR, platelet-to-lymphocyte ratio; RBC, red blood cell; WBC, white blood cell

Characteristics	K6 score	AIS score	CES-D score
Age	ρ= –0.1822 (p<0.0001)	ρ= –0.0917 (p<0.0001)	ρ= –0.0391 (p<0.0001)
Daily tobacco use	ρ= 0.0218 (p<0.0001)	ρ= –0.0299 (p<0.0001)	ρ= 0.0321 (p<0.0001)
BMI	ρ= –0.0531 (p<0.0001)	ρ= –0.0330 (p<0.0001)	ρ= 0.0124 (p=0.0026)
RBC count	ρ= –0.0333 (p<0.0001)	ρ= –0.0501 (p<0.0001)	ρ= –0.0320 (p<0.0001)
Hemoglobin	ρ= –0.0733 (p<0.0001)	ρ= –0.0913 (p<0.0001)	ρ= –0.0516 (p<0.0001)
Hematocrit	ρ= –0.0728 (p<0.0001)	ρ= –0.0882 (p<0.0001)	ρ= –0.0539 (p<0.0001)
Platelet count	ρ= 0.0570 (p<0.0001)	ρ= 0.0352 (p<0.0001)	ρ= 0.0324 (p<0.0001)
WBC count	ρ= –0.0087 (p=0.0321)	ρ= –0.0159 (p<0.0001)	ρ= 0.0212 (p<0.0001)
Neutrophil count	ρ= 0.0100 (p=0.0132)	ρ= –0.0035 (p=0.3803)	ρ= 0.0294 (p<0.0001)
Lymphocyte count	ρ= –0.0300 (p<0.0001)	ρ= –0.0251 (p<0.0001)	ρ= –0.0053 (p=0.1956)
Monocyte count	ρ= –0.0290 (p<0.0001)	ρ= –0.0331 (p<0.0001)	ρ= 0.0136 (p=0.0009)
Eosinophil count	ρ= –0.0115 (p=0.0047)	ρ= –0.0091 (p=0.0245)	ρ= 0.0011 (p=0.7802)
Basophil count	ρ= 0.0026 (p=0.5279)	ρ= –0.0020 (p=0.6286)	ρ= –0.0026 (p=0.5229)
NLR	ρ= 0.0333 (p<0.0001)	ρ= 0.0172 (p<0.0001)	ρ= 0.0308 (p<0.0001)
MLR	ρ= –0.0030 (p=0.4660)	ρ= –0.0121 (p=0.0028)	ρ= 0.0187 (p<0.0001)
PLR	ρ= 0.0648 (p<0.0001)	ρ= 0.0456 (p<0.0001)	ρ= 0.0272 (p<0.0001)

Logistic regression models with K6 ≥13, AIS ≥6, and CES-D ≥16

Based on the findings of the bivariate correlation analyses, age, sex, daily tobacco use, and BMI were used as explanatory variables in addition to Hb levels in the subsequent logistic regression analyses. In the logistic regression models, the K6, AIS, and CES-D scores were used as dependent variables using the aforementioned cutoff levels. The results obtained from the 62,796 volunteers are summarized in Table 2. A significant aOR of the Hb level was observed with the outcomes of CES-D score ≥16 (aOR [IQR], 0.9647 [0.9487-0.9810], p<0.0001), but not with K6 score ≥13 (aOR, 0.9785 [0.9496-1.0083], p=0.1563) and AIS score ≥6 (aOR, 0.9863 [0.9694-1.0035], p=0.1171).

**Table 2:** Logistic regression analyses for each mental disturbance score among the 62,796 participants Three types of logistic regression analyses were performed on 62,796 participants. Each model used one of the three self-reported mental health-related indices as the dependent variable. aOR, adjusted odds ratio; CI, confidence interval; VIF, variance inflation factor * Adjusted OR for a one-unit (1.0) increase, not range OR.

Characteristics	aOR	95% CI	p-value	VIF
Logistic regression model for K6 score ≥13				
Sex (male)	0.5594	0.5072 – 0.6170	<0.0001	1.5876
Age *	0.9621	0.9596 – 0.9647	<0.0001	1.0501
Tobacco per day *	1.0232	1.0180 – 1.0284	<0.0001	1.1470
BMI *	1.0183	1.0090 – 1.0276	0.0001	1.0538
Hemoglobin *	0.9785	0.9496 – 1.0083	0.1563	1.5387
Logistic regression model for AIS score ≥6				
Sex (male)	0.6070	0.5767 – 0.6389	<0.0001	1.5876
Age *	0.9933	0.9916 – 0.9950	<0.0001	1.0501
Tobacco per day *	1.0049	1.0017 – 1.0082	0.0029	1.1470
BMI *	1.0078	1.0024 – 1.0133	0.0048	1.0538
Hemoglobin *	0.9863	0.9694 – 1.0035	0.1171	1.5387
Logistic regression model for CES-D score ≥16				
Sex (male)	0.6516	0.6199 – 0.6849	<0.0001	1.5876
Age *	0.9834	0.9818 – 0.9850	<0.0001	1.0501
Tobacco per day *	1.0149	1.0118 – 1.0179	<0.0001	1.1470
BMI *	1.0137	1.0083 – 1.0190	<0.0001	1.0538
Hemoglobin *	0.9647	0.9487 – 0.9810	<0.0001	1.5387

Subgroup analyses by sex

Next, the same logistic regression models were applied to both the male and female populations. The results obtained from 23,663 male participants are summarized in Table 3. The significant aOR of the Hb level was confirmed with AIS score ≥6 (unit aOR [IQR], 0.9462 [0.9190-0.9743], p=0.0002) and CES-D score ≥16 (unit aOR, 0.9499 [0.9237-0.9767], p=0.0003) were confirmed. The results for the 39,133 female participants are summarized in Table 4. A significant aOR of the Hb level was confirmed with a CES-D score ≥16 (unit aOR [IQR], 0.9707 [0.9501-0.9918], p=0.0066). The statistical significance with K6 score ≥13 was marginal (unit aOR, 0.9655 [0.9316 - 1.0007], p=0.0546).

**Table 3:** Logistic regression analyses for each mental disturbance score among the 23,663 male participants Sensitivity analyses were performed on 23,663 male participants. The statistically significant aORs were reproduced both with AIS score (≥6) and CES-D score (≥16). * Adjusted OR for a one-unit (1.0) increase, not range OR. aOR, adjusted odds ratio; VIF, variance inflation factor

Characteristics	aOR	95% CI	p-value	VIF
Logistic regression model for K6 score ≥13				
Age *	0.9481	0.9433 – 0.9529	<0.0001	1.0866
Tobacco per day *	1.0103	1.0036 – 1.0170	0.0030	1.0572
BMI *	1.0088	0.9895 – 1.0285	0.3765	1.0480
Hemoglobin *	0.9480	0.8938 – 1.0054	0.0755	1.1079
Logistic regression model for AIS score ≥6				
Age *	0.9846	0.9814 – 0.9877	<0.0001	1.0866
Tobacco per day *	0.9969	0.9930 – 1.0007	0.1140	1.0572
BMI *	1.0104	0.9996 – 1.0213	0.0586	1.0480
Hemoglobin *	0.9462	0.9190 – 0.9743	0.0002	1.1079
Logistic regression model for CES-D score ≥16				
Age *	0.9812	0.9782 – 0.9841	<0.0001	1.0866
Tobacco per day *	1.0088	1.0053 – 1.0124	<0.0001	1.0572
BMI *	1.0021	0.9919 – 1.0125	0.6832	1.0480
Hemoglobin *	0.9499	0.9237 – 0.9767	0.0003	1.1079

**Table 4:** Logistic regression analyses for each mental disturbance score among the 39,133 female participants Sensitivity analyses were performed for the 39,133 female participants. This time, the significant aORs were not reproduced with AIS score (≥6) and CES-D score (≥16). * Adjusted OR for a one-unit (1.0) increase, not range OR. aOR, adjusted odds ratio; VIF, variance inflation factor

Characteristics	Adjusted OR	95% CI	p-value	VIF
Logistic regression model for K6 score ≥13				
Age *	0.9681	0.9650 – 0.9712	<0.0001	1.0552
Tobacco per day *	1.0458	1.0373 – 1.0544	<0.0001	1.0397
BMI *	1.0180	1.0075 – 1.0286	0.0008	1.0307
Hemoglobin *	0.9655	0.9316 – 1.0007	0.0546	1.0318
Logistic regression model for AIS score ≥6				
Age *	0.9971	0.9951 – 0.9991	0.0060	1.0552
Tobacco per day *	1.0271	1.0207 – 1.0336	<0.0001	1.0397
BMI *	1.0056	0.9993 – 1.0119	0.0841	1.0307
Hemoglobin *	0.9950	0.9734 – 1.0171	0.6554	1.0318
Logistic regression model for CES-D score ≥16				
Age *	0.9845	0.9826 – 0.9865	<0.0001	1.0552
Tobacco per day *	1.0340	1.0276 – 1.0404	<0.0001	1.0397
BMI *	1.0175	1.0113 – 1.0238	<0.0001	1.0307
Hemoglobin *	0.9707	0.9501 – 0.9918	0.0066	1.0318

Logistic regression analyses with other blood cell profiles

Similar logistic regression analyses were performed for each of the WBC and platelet counts among the 62,796 participants, using age, sex, daily tobacco use, and BMI, together with each of these blood cell count data as covariates. When the K6 score ≥13 was used as the outcome, neither WBC (p=0.8447) nor platelet count (p=0.4588) was significantly associated with the outcome. When the AIS score ≥6 was used as the outcome, neither WBC (p=0.2007) nor platelet count (p=0.9087) was significantly associated with the outcome. When the CES-D score ≥16 was used as the outcome, both the WBC count (p=0.0206) and platelet count (p=0.0631) showed a marginally significant aOR with the outcome.

The same analyses were performed for the NLR, MLR, and PLR. Sex, age, daily tobacco use, and BMI were used as explanatory variables together with each of these ratios. The NLR showed a significant aOR with all K6 score ≥13 (unit aOR, 1.1031 [1.0641-1.1434], p<0.0001), AIS score ≥6 (unit aOR, 1.0543 [1.0315-1.0777], p<0.0001), and CES-D score ≥16 (unit aOR, 1.0936 [1.0706-1.1172], p<0.0001). The MLR showed a significant aOR with all K6 score ≥13 (aOR, 4.9235 [3.0358-7.9850], p<0.0001), AIS score ≥6 (aOR, 2.2370 [1.6774-2.9832], p<0.0001), and CES-D score ≥16 (aOR, 3.5789 [2.7089-4.7284], p<0.0001). The PLR showed a significant aOR with all K6 score ≥13 (unit aOR, 1.0017 [1.0010-1.0023], p<0.0001), AIS score ≥6 (aOR, 1.0006 [1.0002-1.0010], p=0.0042), and CES-D score ≥16 (unit aOR, 1.0011 [1.0008-1.0015], p<0.0001). These statistical significances did not change even when the Hb level was added to the explanatory variables of the logistic regression models.

## Discussion

In this retrospective observational study, using a large population-based medical database, a significant impact of lower Hb levels and elevated NLR, MLR, and PLR on the depressive state and/or sleep disturbance in the general population was observed. These findings did not change after adjusting for age, sex, BMI, and daily tobacco use. In the subgroup analyses, a significant association between lower Hb levels and higher CES-D scores was observed in both males and females but was more prominent in the former. A significant association between lower Hb levels and sleep disturbance was observed in males alone.

Conceivable mechanisms linking lower Hb levels to depression or sleep disturbance may include iron deficiency, although this was not evaluated in the present study. The importance of body iron status has been described as appropriate for emotional behavior and mental health by influencing energy metabolism and neurotransmitter homeostasis in the central nervous system [16]. Based on previous animal experiments, iron plays pivotal roles in brain myelination, monoaminergic systems, and glutamate and γ-aminobutyric acid homeostasis [17-19]. Consequently, iron deficiency can cause various cognitive, emotional, and psychological problems due to impaired iron-related neurochemical circuits. Increased fearfulness has been observed both in animals and humans with poor iron status [20,21]. Iron replacement therapy demonstrated an improvement in the mental development of children [22]. A recent nationwide database analysis from Taiwan revealed that iron deficiency was associated with various mental problems, such as anxiety, depression, and sleep disturbance, and iron replacement therapy was found to decrease the risk of these psychiatric conditions [23]. A recent study in young non-anemic females revealed that even mild iron deficiency may result in central nervous system dysfunction, leading to impaired cognitive endurance and task performance, stimulus-seeking behavior, and mood disorders [24]. Further studies are required to determine the role of iron deficiency in depression and sleep disorders. Whether these mental health problems can be treated with oral iron supplementation requires confirmation.

 This study also found that the NLR, MLR, and PLR were significantly associated with depression and sleep disturbance in the general population after adjusting for demographic background. Possible roles for elevated inflammatory hematological ratios have been proposed as potential prognostic biomarkers in various conditions, such as cardiovascular diseases, cancers, and ischemic stroke [25-29]. Furthermore, possible roles for these ratios in depression and other psychiatric conditions have been proposed, and the findings of the present study are consistent with those of previous reports [30-33]. Another report also demonstrated an elevation in MLR in patients suspected of having psychosomatic disorders compared with healthy volunteers [34]. These findings of elevated inflammatory hematological ratios in miscellaneous mental disorders may offer deeper insights into the mechanisms underlying the development of a depressive state and sleep disturbance in the general population. The potential benefit of measuring the inflammatory hematological ratios in people with mental health problems is further supported by several previous studies demonstrating reduced major depressive symptoms and severity in patients with schizophrenia by using anti-inflammatory therapies [35-37]. Another previous study in patients with major depressive disorder demonstrated higher serum levels of interleukin-6 in non-responders to antidepressant therapy compared to those in responders [38]. These facts collectively indicate that depressive disorders can be derived from heterogeneous mechanisms, including chronic inflammation. Further studies are needed to explore potential therapeutic interventions for individuals with psychiatric conditions showing signs of systemic inflammation.

This study had several limitations. First, serum levels of iron, ferritin, and erythropoietin levels were not evaluated. Therefore, the exact etiology of anemia in each participant (e.g., malnutrition including iron deficiency, chronic kidney disease, chronic inflammation, etc.) could not be determined. Future studies should include additional RBC-related laboratory data, such as iron, ferritin, and erythropoietin (EPO) levels, mean corpuscular volume, and mean corpuscular hemoglobin concentration. Another limitation was that most participants were of Asian ancestry, and the generalizability of the findings to other races and ethnicities remains to be determined. Finally, this study did not investigate the comorbidities that could have influenced the evaluated blood cell profiles. Malnutrition is one of such potential confounders. However, the present study adjusted for the BMI in the logistic regression models, and the potential impact of malnutrition on the findings in this study would be limited. Another conceivable unevaluated comorbidity that may influence the Hb level was sleep apnea syndrome (SAS). In patients with SAS, the apnea-hypopnea index is known to show a significant positive correlation with the hematocrit level [39,40]. A possible theory is that the prolonged condition of hypoxemia during sleep in patients with SAS may stimulate the production of EPO from the kidney [41]. This known relationship between SAS and polycythemia is contrary to the observed relationship between sleep disturbance and low Hb level in this study, making the potential bias derived from SAS also unlikely.

## Conclusions

This study identified the significant effects of lower Hb levels and elevated NLR, MLR, and PLR on depressive states and sleep disturbances in the general population. These associations were significant even after adjusting for potential confounders such as age, sex, BMI, and daily tobacco use. Associations were observed in both males and females but were more prominent in males. Further studies are warranted to determine the mechanisms that link blood cell profiles to mental health problems.
